# Non-dental Effects of Rapid Maxillary Expansion in Growing Children: A Literature Review

**DOI:** 10.7759/cureus.99165

**Published:** 2025-12-13

**Authors:** Faisal S AlSuliman, Yaman Nerabani, Moustafa Alhashemi

**Affiliations:** 1 Department of Orthodontics, King Fahad Medical City, Riyadh, SAU; 2 Department of Cardiology, Aleppo University Hospital, Aleppo, SYR; 3 Faculty of Medicine, University of Aleppo, Aleppo, SYR

**Keywords:** maxillary hypoplasia, non-dental effects, palatal expansion, pediatric dentistry, rapid maxillary expansion, rme

## Abstract

Maxillary hypoplasia is a common malocclusion associated with various clinical conditions, including upper dental crowding, posterior crossbite, narrow palates or high arches, and class II or III malocclusions. These conditions can also have negative non-dental effects in growing children, such as impaired breathing, reduced airway dimensions, obstructive sleep apnea, otological disorders, altered vocal function, and changes in muscle activity. Appliances used to treat these malocclusions may include rapid maxillary expansion (RME), slow maxillary expansion, or mandibular advancement appliances, whether orthodontic or orthopedic. Many studies in the literature compare these devices and highlight their positive effects from a dental perspective; however, few address the non-dental effects on children’s health and quality of life. A thorough and up-to-date review of this literature is essential for healthcare professionals. Therefore, we conducted a comprehensive review to discuss the non-dental effects of RME in pediatric dental patients.

## Introduction and background

Rapid maxillary expansion (RME) is a routine clinical procedure in orthodontics for children and has been used for over 150 years to correct transverse maxillary deficiency. Candidates for RME include patients with dental crowding, posterior crossbite, narrow palates or high arches, and individuals with class II or III malocclusions. The primary purpose of RME is to widen the maxilla in young patients with transverse maxillary constriction and a deep palatal vault [1,2]. This procedure involves using an expansion screw attached to bands on the first premolars and first molars, or in similar placements. The screw is periodically activated daily, causing the mid-palatal suture to open and the maxillary bones to move apart [3]. RME is based on a major, intermittent force system that can lead to dentoalveolar tipping of the posterior teeth [4].

While RME primarily aims to correct dental and skeletal maxillary transverse constrictions, the literature demonstrates its positive effects on breathing, increasing nasopharyngeal airway dimensions, and reducing obstructive sleep apnea (OSA) [5-7]. Several studies indicate that a narrowed maxilla may contribute to the development of more serious breathing disorders, such as OSA in young individuals [8]. RME also helps prevent skeletal asymmetry and disruptions in muscle function [9]. Villano et al. reported improvement in congenital hearing loss in patients who used RME for eight months [10]. Early application of RME may therefore improve breathing, hearing, and muscle function.

Maxillary expansion can be categorized based on the force applied and the frequency of expansion. These include RME, semi-RME, and slow maxillary expansion (SME) [11]. Rabah et al. compared rapid versus SME during early adolescence and found no significant differences in skeletal or dental changes, except for inter-premolar width at the root apex, where the SME group showed greater change. Skeletal palatal width in the molar area also demonstrated a larger increase in the SME group [12]. Children treated with a removable slow maxillary expander experienced less pain and discomfort and encountered fewer difficulties with chewing and swallowing compared to those treated with a bonded rapid maxillary expander [13].

The main objective of this article is to review studies investigating the effects of RME on breathing, airway dimensions, OSA, otological disorders, vocal function, and muscle activity in growing children.

## Review

Effects on breathing and airway dimensions

Recent scientific literature shows significant improvement in many respiratory problems in growing children and adolescents after RME. In the short term, RME promotes enlargement of the dental arches and improvement in mouth breathing [14,15]. Accordingly, six months and one year after RME, nasal airflow was improved in mouth-breathing children [16]. Significant increases in linear maxillary dimensions and nasal cavity size have been associated with improvements in patients’ quality of life [17]. Parents also reported improvements in sleep quality and breathing patterns after RME [18].

An umbrella review of 11 systematic reviews reporting 132 individual studies concluded that RME reduced airway resistance and improved nasal breathing by increasing nasal and oropharyngeal space volumes [19]. However, Matsumoto et al. reported that the decrease in nasal resistance observed at 90 days was not maintained at 30 months after RME [20]. In 2020, Niu et al. presented a review of three-dimensional analyses of the short- and long-term effects of RME on the nasal cavity and upper airway and concluded that RME has a short-term positive impact on expanding the volume of the nasal cavity and the upper portion of the upper airway; however, stability could not be maintained in the long term [21].

The primary volumetric effect of RME is a marked enlargement of the nasal cavity. Husson et al. conducted a study assessing short-term volumetric changes in the oropharyngeal airway of growing Class III maxillary-deficient patients treated with a facemask without expansion. This group was compared with untreated Class III control subjects using low-dose computed tomography. The findings indicated that facemask therapy did not produce significant effects on the oropharyngeal airway beyond those attributable to normal growth [22]. The literature largely indicates that changes in nasopharyngeal and oropharyngeal volumes are statistically insignificant. Consequently, this anatomical increase cannot directly translate into enhanced airway function unless supported by specific evidence [23].

When RME is indicated, it should be performed either during or after treatment of the underlying cause of nasal obstruction. Expansion of the nasal structures through RME can result in immediate improvement in breathing; however, ongoing inflammation of the nasal mucosa may contribute to the recurrence of mucosal hypertrophy [20].

A review by Aziz et al. indicated that RME may have a beneficial effect on nasal septum asymmetry during childhood; however, no significant changes were observed in adolescents with nasal septal defects following RME [24]. This suggests that while RME may be advantageous in younger patients, its effects may be reduced or less noticeable in older individuals with preexisting nasal conditions. Further research is needed to evaluate the long-term effects of RME on nasal structures across different age groups.

Effects on OSA

OSA is a chronic disorder characterized by repeated episodes of partial or complete upper airway obstruction during sleep. These events cause disruptions in breathing, leading to decreased oxygen levels and frequent awakenings, which significantly impair sleep quality [18]. The prevalence of OSA in children ranges from 1.2% to 5.7%, with a higher incidence reported in boys than in girls [25].

Treatment options for OSA include continuous positive airway pressure, surgical procedures, and oral appliances. Orthopedic treatment options for mild to moderate OSA include RME and mandibular advancement appliances (MAAs). Droppelmann et al. found insufficient evidence to conclude that RME or MAAs can fully treat OSA; however, both have been shown to reduce the apnea-hypopnea index (AHI) and alleviate clinical symptoms. RME produced a significant reduction in AHI and an increase in minimum oxygen saturation immediately after active treatment [26]. Although MAAs demonstrated short-term improvements in AHI and oxygen saturation, their effectiveness in treating pediatric OSA remains unclear [27].

RME decreases nasal resistance, facilitating nasal airflow and improving respiratory function. In 2019, a systematic review and meta-analysis evaluated the effects of RME on OSA and hypopnea syndrome in children with sagittal or transverse intermaxillary discrepancies [28]. The review concluded that RME was effective in managing mild to moderate hypopnea and sleep apnea syndrome by improving oximetric parameters. Additionally, RME served as a beneficial adjunct to adenotonsillectomy in severe cases involving pediatric patients with maxillary compression.

Camacho et al. conducted a systematic review and meta-analysis of pediatric patients with OSA who underwent RME. Their results demonstrated a significant reduction in the AHI, decreasing from 8.9 events per hour to 2.7 events per hour within a follow-up period of less than three years [29]. Supporting these findings, Sánchez-Súcar et al. reported a mean AHI reduction of 5.79 events per hour [28]. These results suggest that RME may be an effective intervention for improving sleep apnea symptoms in children.

Early RME using a propulsor universal light appliance significantly improved airway patency in children with Marfan syndrome and has been recommended as a standard treatment for preventing OSA and correcting class II malocclusions [30].

Effects on otological disorders

Maxillary constriction has been suggested to be associated with hearing impairment through its relationship with the Eustachian tubes, middle ear, and mouth breathing [31]. The connection between maxillary expansion and improved hearing may be related to soft tissue changes. Modification of palatal anatomy can affect muscle function by stretching the elevator and tensor veli palatini muscles, thereby supporting proper tympanic membrane and auditory system function [32].

The literature includes numerous studies demonstrating the benefits of RME in improving hearing, particularly in growing children. A systematic review of nine studies reported that eight demonstrated hearing improvements ranging from 2 to 19 dB. The remaining study by Micheletti et al. found no significant differences in hearing following RME or between treatment and control groups [33,34].

In 2016, Kılıç et al. conducted a clinical trial comparing RME with middle ear ventilation tube placement for hearing improvement in children with resistant otitis media with effusion. The study concluded that RME produced effects comparable to ventilation tube insertion in reducing otitis media and improving hearing thresholds. RME was recommended as the initial treatment option for children with maxillary constriction and persistent otitis media with effusion [35]. The rationale for using RME is similar to that of ventilation tube placement for managing Eustachian tube dysfunction. Expansion of the maxilla stretches the muscles responsible for dilating the Eustachian tube, facilitating opening of its pharyngeal orifice and improving function [36].

Rosso et al. reported that conductive hearing loss and middle ear effusion improved significantly during RME and after six months of follow-up [37]. A multicenter randomized controlled trial demonstrated that correction of palatal anatomy through RME significantly improved hearing and middle ear function in patients with normal hearing and mild conductive hearing loss, including those with and without bilateral cleft lip and palate [38]. Nevertheless, further well-designed studies are needed to establish more definitive conclusions.

Effects on vocal function and muscle activity

Voice and speech are complex physiological processes resulting from interactions among the respiratory, laryngeal, and resonator systems [39]. Yurttadur et al. found that RME is safe to apply, as it does not alter vocal quality or resonance [40]. Additionally, RME significantly improved voice quality in non-cleft patients, although no significant changes were observed in patients with bilateral cleft lip and palate [38]. In contrast, De Felippe et al. reported that RME had a detrimental effect on speech [41]. Similarly, Stevens et al. evaluated speech articulation following RME placement and found that speech acceptability declined in all participants after appliance installation [42].

A prospective clinical study evaluated coronal CT scans of 30 subjects (14 boys and 16 girls; mean age, 12.01 ± 0.75 years) before RME (T0) and at the end of the expansion phase (T1) [43]. After RME, all patients demonstrated statistically significant increases in transverse mid-palatal suture dimensions. Anterior CT scans revealed marked expansion of both the nasal cavity and the skeletal base of the maxilla. Significant changes were observed in vocal parameters, including maximum fundamental frequency, jitter, and shimmer. These vocal changes were significantly associated with the widening of the nasal cavity.

In June 2024, a systematic review and meta-analysis including nine studies evaluated whether RME improves masticatory muscle function (masseter and temporalis) in individuals with unilateral posterior crossbite. The review demonstrated improved masseter and temporalis muscle activity following RME [44]. In contrast, a retrospective study reported that surgically assisted RME did not alter superficial electromyographic activity of the masseter and temporalis muscles at T0 and T1 under static conditions in adults with adequate preoperative muscular coordination [45].

## Conclusions

A comprehensive literature review showed that the use of RME in growing children with dental crowding, posterior crossbite, narrow palates or high arches, or class II or III malocclusions has non-dental effects in addition to its beneficial dental effects. It can be concluded that RME significantly improves breathing, airway dimensions, OSA, otological disorders, vocal function, and muscle activity. However, additional high-quality primary studies are needed to confirm these conclusions and to identify other non-dental effects of these appliances on health aspects related to children’s lifestyles.

## References

[1] McNamara JA (2000). Maxillary transverse deficiency. Am J Orthod Dentofacial Orthop.

[2] Agarwal A, Mathur R (2010). Maxillary expansion. Int J Clin Pediatr Dent.

[3] Halicioğlu K, Kiliç N, Yavuz İ, Aktan B (2010). Effects of rapid maxillary expansion with a memory palatal split screw on the morphology of the maxillary dental arch and nasal airway resistance. Eur J Orthod.

[4] Martina R, Cioffi I, Farella M (2012). Transverse changes determined by rapid and slow maxillary expansion - a low-dose CT-based randomized controlled trial. Orthod Craniofac Res.

[5] Bishara SE, Staley RN (1987). Maxillary expansion: clinical implications. Am J Orthod Dentofacial Orthop.

[6] Alexander NS, Schroeder JW Jr (2013). Pediatric obstructive sleep apnea syndrome. Pediatr Clin North Am.

[7] Alam MK, Hajeer MY, Alshammari AH, Alshammari SA, Alenezi ZM (2024). Effect of orthodontic treatment on airway dimensions in patients with obstructive sleep apnea: a prospective study. J Pharm Bioallied Sci.

[8] Felicita AS (2015). Rapid maxillary expansion as a standard treatment for obstructive sleep apnea syndrome: a systematic review. IOSR J Dent Med Sci.

[9] Arat FE, Arat ZM, Acar M, Beyazova M, Tompson B (2008). Muscular and condylar response to rapid maxillary expansion. Part 1: electromyographic study of anterior temporal and superficial masseter muscles. Am J Orthod Dentofacial Orthop.

[10] Villano A, Grampi B, Fiorentini R, Gandini P (2006). Correlations between rapid maxillary expansion (RME) and the auditory apparatus. Angle Orthod.

[11] Bucci R, D'Antò V, Rongo R, Valletta R, Martina R, Michelotti A (2016). Dental and skeletal effects of palatal expansion techniques: a systematic review of the current evidence from systematic reviews and meta-analyses. J Oral Rehabil.

[12] Rabah N, Al-Ibrahim HM, Hajeer MY, Ajaj MA (2022). Evaluation of rapid versus slow maxillary expansion in early adolescent patients with skeletal maxillary constriction using cone-beam computed tomography: a short-term follow-up randomized controlled trial. Dent Med Probl.

[13] Rabah N, Al-Ibrahim HM, Hajeer MY, Ajaj MA, Mahmoud G (2022). Assessment of patient-centered outcomes when treating maxillary constriction using a slow removable versus a rapid fixed expansion appliance in the adolescence period: a randomized controlled trial. Cureus.

[14] Cappellette M Jr, Nagai LH, Gonçalves RM, Yuki AK, Pignatari SS, Fujita RR (2017). Skeletal effects of RME in the transverse and vertical dimensions of the nasal cavity in mouth-breathing growing children. Dental Press J Orthod.

[15] Sakai RH, de Assumpção MS, Ribeiro JD, Sakano E (2021). Impact of rapid maxillary expansion on mouth-breathing children and adolescents: a systematic review. J Clin Exp Dent.

[16] Torre H, Alarcón JA (2012). Changes in nasal air flow and school grades after rapid maxillary expansion in oral breathing children. Med Oral Patol Oral Cir Bucal.

[17] Izuka EN, Feres MF, Pignatari SS (2015). Immediate impact of rapid maxillary expansion on upper airway dimensions and on the quality of life of mouth breathers. Dental Press J Orthod.

[18] Fariña-Vélez M, Parra-Stuardo M, Leal-Hausdorf R (2017). Decreased airway permeability with the use of occlusal stabilization splints in patients with sleep-disordered breathing. A literature review. Rev Clin Periodoncia Implantol Rehabil Oral.

[19] Garrocho-Rangel A, Rosales-Berber MÁ, Ballesteros-Torres A (2023). Rapid maxillary expansion and its consequences on the nasal and oropharyngeal anatomy and breathing function of children and adolescents: an umbrella review. Int J Pediatr Otorhinolaryngol.

[20] Matsumoto MA, Itikawa CE, Valera FC, Faria G, Anselmo-Lima WT (2010). Long-term effects of rapid maxillary expansion on nasal area and nasal airway resistance. Am J Rhinol Allergy.

[21] Niu X, Di Carlo G, Cornelis MA, Cattaneo PM (2020). Three-dimensional analyses of short- and long-term effects of rapid maxillary expansion on nasal cavity and upper airway: a systematic review and meta-analysis. Orthod Craniofac Res.

[22] Husson AH, Burhan AS, Hajeer MY, Nawaya FR (2021). Three-dimensional oropharyngeal airway changes after facemask therapy using low-dose computed tomography: a clinical trial with a retrospectively collected control group. Prog Orthod.

[23] Balasubramanian S, Kalaskar R, Kalaskar A (2022). Rapid maxillary expansion and upper airway volume: systematic review and meta-analysis on the role of rapid maxillary expansion in mouth breathing. Int J Clin Pediatr Dent.

[24] Aziz T, Ansari K, Lagravere MO, Major MP, Flores-Mir C (2015). Effect of non-surgical maxillary expansion on the nasal septum deviation: a systematic review. Prog Orthod.

[25] Koretsi V, Eliades T, Papageorgiou SN (2018). Oral interventions for obstructive sleep apnea. Dtsch Arztebl Int.

[26] Droppelmann-Muñoz T, Carmash-Kretschmar C, Zursiedel-Puentes MI, Traub-Valdés V, Valdés-Kufferat C (2021). Therapeutic alternatives for obstructive sleep apnea syndrome in children with sagittal and transverse intermaxillary anomalies: narrative review. Int J Interdiscip Dent.

[27] Nazarali N, Altalibi M, Nazarali S, Major MP, Flores-Mir C, Major PW (2015). Mandibular advancement appliances for the treatment of paediatric obstructive sleep apnea: a systematic review. Eur J Orthod.

[28] Sánchez-Súcar AM, Sánchez-Súcar FB, Almerich-Silla JM, Paredes-Gallardo V, Montiel-Company JM, García-Sanz V, Bellot-Arcís C (2019). Effect of rapid maxillary expansion on sleep apnea-hypopnea syndrome in growing patients. A meta-analysis. J Clin Exp Dent.

[29] Camacho M, Chang ET, Song SA (2017). Rapid maxillary expansion for pediatric obstructive sleep apnea: a systematic review and meta-analysis. Laryngoscope.

[30] Taddei M, Alkhamis N, Tagariello T, D'Alessandro G, Mariucci EM, Piana G (2015). Effects of rapid maxillary expansion and mandibular advancement on upper airways in Marfan's syndrome children: a home sleep study and cephalometric evaluation. Sleep Breath.

[31] Braun F (1966). A contribution to the problem of bronchial asthma and extension of the palatine suture. Rep Congr Eur Orthod Soc.

[32] Kemaloğlu YK, Kobayashi T, Nakajima T (2000). Associations between the Eustachian tube and craniofacial skeleton. Int J Pediatr Otorhinolaryngol.

[33] Fagundes NC, Rabello NM, Maia LC, Normando D, Mello KC (2017). Can rapid maxillary expansion cause auditory improvement in children and adolescents with hearing loss? A systematic review. Angle Orthod.

[34] Micheletti KR, de Mello JA, de Almeida Barreto Ramos SR, Scheibel PC, Scheibel GG, Ramos AL (2012). Effects of rapid maxillary expansion on middle ear function: one-year follow-up. Int J Pediatr Otorhinolaryngol.

[35] Kılıç N, Yörük Ö, Kılıç SC, Çatal G, Kurt S (2016). Rapid maxillary expansion versus middle ear tube placement: comparison of hearing improvements in children with resistance otitis media with effusion. Angle Orthod.

[36] Kilic N, Kiki A, Oktay H, Selimoglu E (2008). Effects of rapid maxillary expansion on conductive hearing loss. Angle Orthod.

[37] Rosso C, Colletti L, Foltran M (2022). Effects of rapid maxillary expansion on hearing loss and otitis media in cleft palate children. Eur Arch Otorhinolaryngol.

[38] Singh H, Maurya RK, Sharma P, Kapoor P, Mittal T, Atri M (2021). Effects of maxillary expansion on hearing and voice function in non-cleft lip palate and cleft lip palate patients with transverse maxillary deficiency: a multicentric randomized controlled trial. Braz J Otorhinolaryngol.

[39] Petrovic-Lazic M, Jovanovic N, Kulic M, Babac S, Jurisic V (2015). Acoustic and perceptual characteristics of the voice in patients with vocal polyps after surgery and voice therapy. J Voice.

[40] Yurttadur G, Bascıftcı FA, Ozturk K (2017). The effects of rapid maxillary expansion on voice function. Angle Orthod.

[41] De Felippe NL, Da Silveira AC, Viana G, Smith B (2010). Influence of palatal expanders on oral comfort, speech, and mastication. Am J Orthod Dentofacial Orthop.

[42] Stevens K, Bressmann T, Gong SG, Tompson BD (2011). Impact of a rapid palatal expander on speech articulation. Am J Orthod Dentofacial Orthop.

[43] Bilgiç F, Damlar İ, Sürmelioğlu Ö, Sözer ÖA, Tatlı U (2018). Relationship between voice function and skeletal effects of rapid maxillary expansion. Angle Orthod.

[44] Nunes GP, Morabito MJ, Nunes LP (2024). Exploring the potential of rapid maxillary expansion and masticatory muscle activity in unilateral posterior crossbite. J Clin Exp Dent.

[45] Abate A, Lanteri V, Marcolongo L, Solimei L, Maspero C (2023). Evaluation of masticatory muscles in adult patients with maxillary hypoplasia treated with surgically assisted rapid maxillary expansion (SARME): a retrospective study. J Clin Med.

